# Telehealth in Neurodegenerative Diseases: Opportunities and Challenges for Patients and Physicians

**DOI:** 10.3390/brainsci11020237

**Published:** 2021-02-13

**Authors:** Fabiola De Marchi, Elena Contaldi, Luca Magistrelli, Roberto Cantello, Cristoforo Comi, Letizia Mazzini

**Affiliations:** 1 Department of Neurology and ALS Centre, Traslational Medicine, University of Piemonte Orientale, Maggiore della Carità Hospital, 28100 Novara, Italy; roberto.cantello@med.uniupo.it (R.C.); letizia.mazzini@uniupo.it (L.M.); 2 PhD Program in Medical Sciences and Biotechnology, University of Piemonte Orientale, 28100 Novara, Italy; contaldie@yahoo.it; 3 Department of Neurology and Movement Disorders Centre, Traslational Medicine, University of Piemonte Orientale and “Maggiore della Carità” University Hospital, 28100 Novara, Italy; magis.luca@gmail.com (L.M.); cristoforo.comi@med.uniupo.it (C.C.); 4 PhD Program in Clinical and Experimental Medicine and Medical Humanities, University of Insubria, 21100 Varese, Italy

**Keywords:** neurodegeneration, neurology, telehealth, telemedicine, Alzheimer’s disease, Parkinson’s Disease, Amyotrophic Lateral Sclerosis

## Abstract

Telehealth, by definition, is distributing health-related services while using electronic technologies. This narrative Review describes the technological health services (telemedicine and telemonitoring) for delivering care in neurodegenerative diseases, Alzheimer’s disease, Parkinson’s Disease, and amyotrophic lateral Sclerosis, among others. This paper aims to illustrate this approach’s primary experience and application, highlighting the strengths and weaknesses, with the goal of understanding which could be the most useful application for each one, in order to facilitate telehealth improvement and use in standard clinical practice. We also described the potential role of the COVID-19 pandemic to speed up this service’s use, avoiding a sudden interruption of medical care.

## 1. Introduction

Neurodegenerative diseases (NDDs) are a heterogeneous group of debilitating and incurable diseases that currently affect >30 million individuals worldwide with devastating consequences for patients and their families. NDDs are characterized by the progressive degeneration of the central nervous system’s structure and function, either due to unknown causes with an idiopathic mechanism or, rarely, to a genetic disorder. NDDs include a large group of patients and they comprehend both common and uncommon diseases with Alzheimer’s disease (AD), Parkinson’s disease (PD), and Amyotrophic Lateral Sclerosis (ALS), among the most challenging [1]. The most evident risk factor for developing this condition is aging and, with the increase of the population average age, the prevalence of NDDs is remarkably increasing [2,3]. This increase leads to an enormous burden on healthcare systems and national economies, both in terms of direct and indirect costs [4,5,6,7].

Over disease progression, NDDs are characterized by a continuous decline of the motor and/or cognitive functions, which makes travel to the medical centers stressful and laborious for patients and caregivers. Besides, the lack of adequate transport, the residence in rural areas, and the limited economic funding can worsen the problem. Consequently, patient and clinician contact becomes highly troublesome in terms of care, monitoring, and intervention. Consequently, it is precisely in this scenario that a telehealth and telemedicine approach may be a useful tool for facing this challenge. By definition, telehealth is the distribution of health-related services using electronic technologies and, in this set, it can improve the continuity of care in patients with chronic NDDs [8]. Telehealth has several facets: telemedicine, telecoaching, and telecare (Table 1).

Despite a theoretical possibility for applying a telehealth approach in several domains, this service’s use is still limited; however, steps have been taken during the recent COVID-19 pandemic, when the health service for chronic patients was suddenly interrupted. Indeed, except for visits with urgent characteristics, the out-patient follow-up visits have been immediately suspended, inevitably generating a sense of loss and abandonment due to the lack of dedicated medical and psychological support. In this context, the need to switch to alternatives types of care, including telemedicine and telehealth, is becoming mandatory in preventing a more significant functional decline.

This narrative review aims to analyze and discuss the primary experience and applications of this approach in the three most represented NDDs (AD, PD, ALS) in order to facilitate telehealth improvement and use in standard clinical practice, highlighting the strengths and weaknesses, with the goal of understanding which could be the most useful application for each one. The present review particularly emphasizes the management of telehealth resources before the COVID-19 outbreak, underlining how they changed with the ongoing pandemic emergency.

## 2. Telemonitoring in Neurodegenerative Diseases

### 2.1. Alzheimer’s Disease

Dementia represents one of the most frequent chronic disabling conditions in the elderly population. It has been estimated that, by 2050, approximately 152 million people will have dementia [9]. AD is the most common cause of dementia, followed by fronto-temporal dementia (FTD), Lewy body dementia (LBD), and dementia after brain injuries (i.e., trauma, stroke). Besides the different molecular pathways involved, patients present with cognitive decline or behavioral changes that progress until their total dependence, which often does not have adequate formation and help. Furthermore, these patients often live in rural areas and have difficulties in reaching the leading centers to be in touched with their doctors and perform regular follow-up visits.

The validity of telemedicine for managing patients with dementia has already been investigated and applied. Notably, telemedicine confirmed its feasibility in monitoring patients’ disease course (thanks to the administration of cognitive tests), allowing strict, though virtual, contact between patients and the clinicians. This aspect recently gained importance, since, due to the recent pandemic caused by the Sars-CoV-2, regular visits were interrupted, and telemedicine was confirmed as a good tool to monitor these patients’ clinical progression, as outlined above [10]. Using self-administered questionnaires, telemedicine has been generally evaluated as an accessible and useful tool by both patients and caregivers, who, in some cases, preferred to continue the clinical follow-up through this instrument. Notwithstanding the above, in some cases, telemedicine may represent a challenge, since not all patients own or can use informatics platforms or are not commonly accessible from the long-term care clinics where patients are often admitted when they are no more independent.

The use of telemedicine in dementia was investigated while considering several aspects, ranging from diagnosis to treatment. Firstly, several studies have evaluated the validity of cognitive tests, which are commonly used during face-to-face visits, when administered using video platforms. Mini Mental State Examination (MMSE) and the Montreal Cognitive Assessment (MoCA), which are commonly used in the clinical setting, but also more complete and complex tests analyzing several cognitive domains, also appeared to be valid when performed using a telemedicine interface [11]. Furthermore, Wadsworth and colleagues [12] demonstrated that commonly used cognitive tests, when performed through telemedicine, could distinguish between patients with and without dementia. Hwang K et al. [13] demonstrated that the virtual interpretation of tests score is also cheaper than the face-to-face one. It is mainly observed in their preliminary cost analysis, a saving of approximately 250,000 Australian Dollars per year (when considering a medium spare of seven dollars per session).

Together with cognitive problems, patients often have pathological behaviors and depression, which may represent one of the current symptoms or may complicate clinical management in the advanced phases. In recent work, Lindauer and colleagues [14] demonstrated that telemedicine platforms represent feasible tools for investigating cognitive domains and behavior and mood items in AD patients evaluated while using the revised memory and behavior problems checklist (RMBPC). Remarkably, the scores obtained using virtual platforms were superimposable to the face-to-face ones.

Another important aspect of dementia management is related to pharmacological treatment. Generally, clinicians decide whether to start or discontinue specific drugs according to clinical course and test scores. Cheong et al. [15] showed that patients followed with telemedicine had longer treatment compliance and, consequently, duration than patients followed in clinics (26.6 and 14.6 months). Furthermore, the authors showed that the use of telemedicine and low Clinical Dementia Rating scores in their rural cohort represented independent predictive factors of longer treatment duration. Several factors may be implied, such as the lower fees of virtual visits, the lack of local specialists, and the higher levels of care that is perceived by the patients.

Telemedicine may also be implied in patients’ cognitive rehabilitation through virtual or augmented reality and serious games. Few studies are now available with positive results, even though the need for specialized figures and the platforms’ high costs may limit the accessibility to these instruments.

Because dementia is an age-related condition, demented subjects often suffer from several comorbidities, requiring more clinical cures and it may lead patients to the emergency department (ED) for acute illness [16]. Telemedicine has been demonstrated as a useful tool in reducing access to the ED. Particularly, Gillespie et al. [17] demonstrated in a group of 201 patients with dementia reducing 24% of the access to ED. Of note, in this work, the authors focused on patients living in the senior living communities (SLC), thus underlying how these particularly fragile patients may benefit from telemedicine to get in touch with their specialists and have faster solutions to their health problems.

Caregivers play an indispensable role in the daily life of patients with dementia. Video platforms have also been used to follow caregivers’ activities and address them in patients’ long-term management. Williams et al. [18], as part of the FamTechCare project, randomly assigned caregivers to video platforms or phone call intervention. Both of the instruments were revealed to be useful and they were appreciated by caregivers, especially those living with mild-moderate demented patients, and the results were not related to age, gender, and relationship. The same project [17] also showed promising results in the education of caregivers evaluated using the Short Sense of Competence Questionnaire (SSCQ): just after three months of video sessions, they showed a more significant gain of competencies than the control group, along with a significant reduction of depressive symptoms. Besides these positive aspects, some studies revealed how, somehow unexpected, caregivers’ compliance decreases as the video follow-up proceeds, thus opening to question whether these protocols may be revised and improved.

Table 2 summarizes the most relevant studies on AD and telemedicine.

### 2.2. Parkinson’s Disease

PD is a neurodegenerative disorder that is clinically characterized by a large number of motor (mainly bradykinesia, resting tremor, rigidity, and postural instability) and nonmotor features (autonomic dysfunction, cognitive/neurobehavioral abnormalities, sleep disorders, and sensory disturbances, such as anosmia, paresthesias, and pain) [29]. Severe motor and cognitive disability may occur as PD progresses to more advanced stages. Some patients may be unable to travel long distances, such as regular follow-up visits at tertiary medical centers.

Telemedicine involves the remote delivery of health care services. Because PD is largely visually assessed and, therefore, well suited to telemedicine, this approach can be a powerful resource for evaluating and managing patients. In order to improve current models of care, telemedicine should reliably assess motor features, and this achievement can be obtained through different devices: Ferreira et al. [30] used an objective system (SENSE-PARK System), which allowed through wearable sensors the quantitative and continuous monitoring of 22 PD patients. Further applications were examined in motor fluctuations: a study [31] confirmed the clinical validity of an algorithm that is able to detect and quantify leg dyskinesias while using a single ankle-worn sensor. The possibility to efficiently capture PD motor features can be useful for identifying potential candidates for advanced therapies: for example, a study from Heldman et al. [32] found that advanced therapy referral rate was significantly higher for patients with remote monitoring reports available as compared to standard care alone (63.6% versus 11.8%, *p*-value < 0.01). Nevertheless, immediate concerns regarding the validity of distant clinical examination have also been raised, since neither rigidity nor balance can be remotely assessed. Additionally, other potentially crucial parts of the neurological exam, such as reflexes or eye movements, are harder to evaluate.

The first studies on telemedicine have mainly focused on this issue: Samii and colleagues [33] reported a three-year use of telemedicine to deliver follow-up care in 34 PD patients, and adequate motor Unified Parkinson’s Disease Rating Scale (UPDRS) measurements were proved when using enhanced video quality. The reliability of a modified UPDRS removing rigidity and retropulsion items as compared to the standard motor scale was also confirmed in a secondary analysis of the CALM-PD study [34]. Besides, when considering travel and lodging costs, considerable resource savings could be achieved [33]. Cubo et al. confirmed the cost-effectiveness of home-based motor monitoring plus standard in-office visits compared to in-office visits alone in 35 PD patients [35]: the home-based care model was cost-effective in terms of functional status, motor impairment, and motor complications, as evaluated by UPDRS II, III, and IV.

In the view of an integrated model of care, some studies have also evaluated the telemedicine economic impact. For example, Dorsey et al. [36] examined the feasibility, effectiveness, and economic advantages of this approach: the authors performed a randomized controlled trial in 20 patients, 11 with in-person visits and nine with specialist care via telemedicine. Each telemedicine visits saved participants, on average, 100 miles of travel, with a relevant economic value. However, life quality change did not differ between the two groups (*p*-value = 0.61). Similar results regarding the quality of life were confirmed in a one-year large, randomized, and controlled trial that was published by Beck et al. [37]: usual care and usual care supplemented by four virtual visits via video conferencing were compared in a cohort of 195 patients. Even though there was consistent economic convenience in the telemedicine group, quality of life did not improve in those receiving virtual house calls. Conversely, a study conducted by Durner et al. [38] found, in a group of 50 PD patients provided with a 24/7 live stream telemedicine home treatment service, that, after one year, there was a significant improvement in Parkinson’s Disease Questionnaire 39 (PDQ39) scores, but not in UPDRS, MMSE, or Hoehn & Yahr (H&Y) scores.

One of the earliest applications of telemedicine as part of an integrated model of care was related to fragile and advanced PD patients: Biglan et al. [39] used live teleconference technology to provide care to a patient residing in a remote nursing home over eight months, which resulted in an improvement in motor and cognitive symptoms and satisfaction from the patient’s perspective. The feasibility of providing care via telemedicine for patients residing in nursing homes was also considered in a randomized controlled trial [40]: the participants were randomized to receive telemedicine care or their usual care, and three telemedicine visits were performed over six months. Patients receiving telemedicine completed almost all of the scheduled visits and showed significant improvement in the quality of life and motor performance. Moreover, when considering the remote evaluation of advanced-stage PD patients, cognitive impairment is also highly likely to occur. It may represent a relevant nonmotor feature: a pilot study from Abdolahi and colleagues [34] tested whether, in 17 individuals with movement disorders, the MoCA examinations could be remotely assessed via web-based video conferencing confirming the feasibility of this approach.

If telemedicine is a valuable resource for patients who cannot perform regular follow-up visits because of long travel distances or significant motor and nonmotor deterioration, the management of intermediate PD stages should be addressed. Marzinzik and colleagues [41] analyzed data from 78 patients that were involved in an integrated care program (ICP). Patients sent home-made video recordings to the treating team via the Internet and, during the 30-day evaluation, an average of 3.2 videos per day was sent. After the ICP conclusion, the UPDRS score was significantly lower than baseline, and the questionnaire’s information showed the overall acceptance and practicability of this method. Another strength of ICP’s use is the consistent schedule of recordings, which can better convey the dynamics of motor fluctuations.

Even though many advantages can be observed, telemedicine programs’ implementation and use have raised concerns regarding the doctor–patient relationship. Some studies have evaluated patients’ perception: Qiang JK and Marras [42] administered to 34 users and 103 non-users of telemedicine services a satisfaction questionnaire. 29/34 users were interested in continuing with telemedicine, and non-users (55/103) were interested in using telehealth, either partially or wholly replacing in-person visits. Among the predictors of telemedicine interest, the authors found that the most relevant ones were lower H&Y stage and longer travel time. Interest in the use of telemedicine was also confirmed in a national randomized controlled trial that was conducted by Dorsey and colleagues in 2016 [43]: during recruitment, 11.734 individuals visited the study’s website, and 927 individuals submitted electronic interest forms, which indicated high interest in receiving remote care. Another survey [44] confirmed the high interest of patients in telemedicine: the main advantages included access to specialists (62%), convenience (60%), and time savings (59%). Therefore, these studies suggest that the doctor–patient relationship is not affected by telemedicine.

Other intriguing developments of telemedicine include speech and physical rehabilitation. Howell et al. [45] proved that delivering the Lee Silverman Voice Treatment (LSVT) by web camera allowed for similar beneficial outcomes when compared to face-to-face treatment in three PD patients. Similar non-inferiority results in subjects treated remotely as compared to conventional in-person LSVT were also found in other studies [46,47]. Moreover, speech assessment can be reliably performed online: Constantinescu and colleagues [48] evaluated, in 61 patients, the level of agreement between an online and face-to-face assessment of hypokinetic dysarthria by two speech-language pathologists and found that, for the majority of parameters, there were no significant differences between the two approaches.

Regarding telerehabilitation, a randomized controlled trial [49] compared, in 76 patients, the treatment with virtual reality (VR) rehabilitation and in-clinic sensory integration balance training (SIBT), and found that, in the VR group, there was a significant improvement in the Berg Balance Scale. Another trial [50] compared 20 patients that were randomized to receive either telecoach-assisted exercise (TAE) or self-regulated exercise (SRE). Both of the groups received the same eight-week exercise prescription with combined strength and aerobic exercise, and the authors found that the TAE participants achieved more robust attendance (99.2%) when compared to SRE participants.

Moreover, a wide variety of nonmotor symptoms may benefit from telemedicine, such as depression and anxiety. A randomized controlled trial [51] proved the feasibility of telephone-administered Cognitive Behavioral Therapy (CBT) to tackle these nonmotor features. Another recent trial [52] further confirmed these results: 72 PD patients were randomized to telephone-based cognitive-behavioral treatment (T-CBT) or treatment as usual. The Hamilton Depression Rating Scale score improved significantly in T-CBT, with a persistent benefit over a six-month follow-up (*p*-value < 0.01). Another study [53] explored both the feasibility and patients’ satisfaction with telepsychiatry services of 33 PD patients who completed 119 telepsychiatry and 62 in-person visits: patients were overall satisfied, even though some technical aspects needed further optimization.

Another relevant PD management issue is related to advanced therapies: a study that was performed by Willows and colleagues [54] evaluated levodopa-carbidopa intestinal gel (LCIG) home titration in 14 patients via telemedicine. The median time that was required for dosage adjustment was lower than hospital titration, with both patients and health practitioners’ satisfaction. Additionally, when considering Deep Brain Stimulation (DBS), telehealth resources have been proved beneficial: the experience of the Ontario Telemedicine Network [55] confirmed in 141 patients retrospectively analyzed the feasibility of telemedicine, which provided adequate interventions allowing, at the same time, a significant reduction in the burden and costs of traveling. Another study [56] explored, in six patients, the feasibility of a technological system (which included a kinematic sensor able to detect motor symptoms) for remote real-time control of apomorphine pumps: this approach was acceptable for three patients and facilitated the initial and long-term dose adjustment.

Lately, the impact of the COVID-19 pandemic has led the scientific community to redefine and improve telemedicine’s role in the healthcare system: for instance, recent guidelines and recommendations [57,58] have highlighted the importance of telemedicine in substituting outpatients’ visits. Expanding telehealth could reduce person-to-person contact, thus lowering the risk of exposure for patients. Substantial innovations are currently being implemented, and the Telemedicine Study Group of the International Parkinson and Movement Disorders Society has updated a guide to telemedicine to tackle these recent challenges (https://www.Movementdisorders.Org/MDS/About/Committees--Other-Groups/Telemedicine-in-Your-Movement-Disorders-Practice-A-Step-by-Step-Guide.Htm (accessed on 12 February 2021)). Various recommendations are being provided, particularly in the management of advanced therapies [59,60,61] and rehabilitation [62,63]. Together with new encouraging models of care (https://www.epda.eu.com/latest/news/parkinson-italia-calls-for-permanent-italian-tele-medicine-service-for-people-with-parkinsons/ (accessed on 12 February 2021) and [58]), concerns about telemedicine becoming a new gold standard of treatment have also been raised [64]: therefore, additional research is warranted to elucidate the benefits and limitations of telemedicine in PD.

Table 3 summarizes the most relevant studies on PD and telemedicine.

### 2.3. Amyotrophic Lateral Sclerosis

ALS is a rare progressive neurodegenerative disease that is caused by the degeneration of upper and lower motoneurons, leading to muscle paralysis and respiratory failure. Currently, there is no cure for ALS, but a specialized multidisciplinary approach has been shown to extend survival and improve the quality of life in patients with ALS, providing a coordinated interdisciplinary team that is able to take charge of patients’ complex needs and become the standard of care. Subsequently, as a care model that is able to keep pace based on the new understanding of this disease, the multidisciplinary team has expanded accordingly, including new healthcare providers and using new technologies [66]. However, this setting is only available in specialized centers, which may be far from the patient’s home, and/or difficult to reach in the disease’s late stages due to functional limitation. Recently, telemedicine, which has received little attention for many years, is proving to be a handy tool in overcoming these difficulties, slowly becoming part of the fundamental features that are offered by multidisciplinary centers [67].

The first study that was published on tele-treatment of patients with ALS dates back to almost 20 years ago [68], intending to discuss disease course and rehabilitation treatment options, observing that tele-treatment was especially suitable for discussing the practical issues, but psychosocial and emotional issues still needed to be discussed during traditional face-to-face contact. However, despite encouraging preliminary results, for many years, this method has not been fully exploited, mostly from European centers.

In 2017, Van De Rijn et al. published the results of the TelePALS study [69]. This study was a retrospective chart review of all patient encounters via video televisits at the MGH ALS clinic (2014–2016), where video televisits were used as a supplement of standard clinical follow-up for patients evaluated at least once in the ALS center. In this study, the authors highlighted that video televisits are convenient for ALS patients, saving time and burden, and evaluating the disease progression in all disease stages. In addition, the same group reported a marked adjusted cost-savings for patients and institutions with telemedicine [70].

A retrospective cohort study analyzed the quality of telemedicine care as compared to traditional ones, observing a higher probability of remaining stable, which means a significantly lower risk of disease progression for patients receiving telemedicine (*p*-value = 0.03) [71]. From a phycological/satisfaction point of view, telemedicine looks like a useful tool, combining good communication and a comfortable interaction and removing travel and stress burdens. In 2019, an Italian study started to use the remote telemedicine evaluation from ALS patients, showing the safety and effectiveness, improving the perceived quality of care and patient satisfaction, as well as reducing in care costs [72].

Recently, novel telehealth approaches were developed to monitor patients and caregivers regarding their condition and capture the variations in physical and emotional areas. The approach that was described by Hobson in 2018 [73] was based on a digital solution where patients, through an app into which patients have to periodically answer several questions about their condition, and the healthcare providers immediately receive the information. As an alternative or added method, the reported potential benefit of this technology included improved communication and care coordination, reassurance, and identification of complications.

A recent Dutch study [74] proposed self-monitoring and messaging, through which patients could self-monitor their well-being (daily report), body weight (weekly), and functional status (monthly). In this population group, the adoption rate was 80%, with a median follow-up of 11 months. The highest adherence was observed in body weight and functional assessment monitoring. An app was also exploited for dietary monitoring in ALS patients, comparing the standard in-person care with counseling supported by an app (“mHealth”). The authors reported that the app-counseling is safe, even being unable to maintain weight significantly better than standard care in ALS patients [75].

Telemonitoring studies have been developed in parallel with telemedicine, in order to supervise clinical parameters at home. The first study with this approach, which was published by an Italian group in 2010 [76,77], described the use of telemedicine-assisted integrated care (TAIC) to monitor the clinical and oxygen variation (supported by a portable pulse oximeter). The study highlights, in addition to a high level of patient and caregiver satisfaction, an important clinical utility, which made it possible to recommend hospitalization to the patient when necessary. In the same year, the use of modem communication for adaptation of Non-Invasive Ventilation (NIV) was tested, showing that telemonitoring reduces health care utilization and in-hospital admissions with favorable implications on costs and functional status [78,79]. In 2019, Ando et al. [80] used a set of self-report questions (the MND OptNIVent question set) to monitor the respiratory functions in patients with NIV weekly, observing the administration of the questionnaire, together with weekly monitoring of ventilator and oximetry, was able to facilitate the maintenance of ventilation and SpO2 levels, despite illness progression.

The management and monitoring of patients with chronic neurodegenerative diseases suddenly changed due to the tragic situation generated by the COVID-19 pandemic [81]. To date, few groups worldwide have reported on the use of various forms of telehealth for monitoring ALS patients during the COVID-19 pandemic. At the beginning of the pandemic, the members of the Northeast ALS (NEALS) Consortium that were surveyed in the U.S. ALS academic medical centers to investigate the possibility to continue to follow-up ALS patients: roughly 50% of patients were unable to see in-person patients, but most could offer video visits [82]. However, to date, only Italian prospective studies have been published. Indeed, three independent Italian groups described their different reality in monitoring ALS patients during the COVID-19 pandemic. Firstly, the group of Logroscino [83] showed that, even with some limitations (e.g., refusal of video interaction), telemedicine was effective and successful for people with ALS, and most patients would like to continue to be included in remote evaluation programs. Similarly, two ALS centers in Northern Italy [84,85], which were located in one of the areas most affected by the pandemic, used the telemedicine service, both via phone call and online platform, for monitoring patients. De Marchi et al. [85,86] reported that the multidisciplinary approach currently used with ALS patients could be reproduced with the telehealth approach. This systemic and periodic monitoring is equally useful for stabilizing the functional and metabolic status and for improving the psychological one. An online Italian self-administered ALS Functional Rating Scale—Revised was developed and used to get feedback about themselves from patients and implement telemedicine monitoring [87].

Table 4 summarizes the most relevant studies on ALS and telemedicine.

## 3. Expert Opinions and Conclusions

This integrated review has shown the achievements of technology from several perspectives, thanks to the ease of application and reproducibility. Nevertheless, to date, the use of technology is still too little developed, even though the COVID-19 pandemic can be considered as an unexpected opportunity to speed up clinical practice approval.

However, telehealth requires a series of considerations and reflections:

(1) legal issues: the protection of data in terms of integrity, access, and security tracking and reporting (as face-to-face out-patient visits) are mandatory. Currently, no global regulation exists, but, in many jurisdictions, there are some limitations: first of all, in several countries, telemedicine visits are not considered to be official, and telehealth is still considered an experimental trial; an added problem is related to the use of this data, where, for privacy and the management of sensitive data, informed consent is required for data collection. Telemedicine technologies have to create encryption between the involved devices, while using virtual private network tunnels for internet connection [89]. In 2015, the European Union increased telemedicine in Europe as a standard medical service, but a set of European rules on telemedicine is still lacking and confusing, and the approach to telemedicine varies in countries [90]. In addition to the legal issues, the reimbursement problem is not secondary: in the U.S., the landscape is evolving, and some insurances provide reimbursement for pre-definite subgroups of patients (e.g., >65 years). In the UE, each single member states are responsible for regulating this question, but to date, many members do not have any legal provisions on telemedicine [90];
(2) personal barriers: doubts regarding acceptance from patients and healthcare providers can slow down the telemedicine application. However, as shown, several studies showed a high level of acceptance from patients, caregivers, and physicians. Patients’ barriers are also related to personal complexity in the use of technology, due to motor limitations, added to the absence of dedicated caregivers and adequate setting. Indeed, sometimes, the mandatory need of a caregiver—both for demented patients due to cognitive limitations and for patients with ALS due to motor limitations—is a critical point, which can inhibit the use of telemedicine. Besides, some patients—as elderly patients and who live in rural areas—do not have available technological devices (e.g., smartphone, tablet, or personal computer) and they have a poor internet connection, which may not allow great televisits, inducing anxiety and discomfort. Improvements in technology, with the creation of a robust information technology infrastructure, can significantly facilitate the practice of telemedicine, particularly in maintaining connectivity and technological resources;
(3) differences with in-person evaluation: although tele-neurological consultations are similar to in-person evaluations, there are significant differences. Information from the virtual visit is only obtained by observing the patient; hence, certain aspects of the examination, such as tone and coordination, may be challenging to assess by visual inspection alone. Moreover, the inconsistencies in responses due to difficulty hearing well or to minor cognitive and behavioral impairment may be more difficult to detect by telemedicine. Hence, techniques for optimizing telemedicine interactions and equipment should be part of a training program for neurologists who participate in telehealth [91]; and,
(4) research and clinical trials: telemedicine can be used in research for several aspects, such as for patients’ screening and recruitment (avoiding stressful travels to the closest recruiting Centre), for informed consent signature, and for patients monitoring [92]. Indeed, telemedicine could reduce the burden of participation, reducing the outpatient visits. However, a new clinical trial design is needed. The virtual patient ‘s assessment will require creating, validating, and collecting self-reported outcome measures, in order to directly obtain an objective score from the patients (avoiding the provider’s video-interpretation), which should be well integrated with the physician measures.

The benefits of telemedicine are several and promising, and it has several possible applications in neurology: we believed that the application of technology in medicine is useful for patients, for healthcare providers, and institutions, allowing easy access to medical care, avoiding stressful travels, and reducing the health care costs and waiting lists. Future research should be addressed in this landscape to improve the quality of service, discover the best-applicated use, and spread it among patients.

## Figures and Tables

**Table 1:** Facets of telehealth.

Domain	Definition
Telehealth	General term to refer to technological health information services, health care education, and health care services
Telemedicine	The term telemedicine refers more specifically to education over a distance of health care services through telecommunications. Telemedicine refers to the use of information technologies and electronic communications to provide remote clinical services to patients (e.g., video consultations, evaluation of medical imaging)
Telecoaching	Telecoaching is coaching delivered over an electronic medium; in the health sector, it means the process of mentoring the use of devices and patient’s management
Telecare	Telecare means a specific method to monitor fragile patients using of alarms, sensors, and other equipment, to help people live independently for a longer time. Telecare comprises assistive technologies and services tailored to individual needs. It monitors activity changes over time and can call for help in emergencies

**Table 2:** **Telemedicine studies in dementia.** Continuous variables are expressed as mean (SD or confidence interval) unless otherwise specified. Abbreviations: U.S.: United States; AD: Alzheimer’s Disease; MCI: mild cognitive impairment; HC: healthy control; VC: videoconference; FTF: face-to-face; MMSE: Mini-Mental State Examination; 15-WVLT: 15-Word Verbal Learning Test; TMT: Trail making test; HIPAA: Health Insurance Portability and Accountability Act; MoCA: Montreal Cognitive Assessment; CDR: Clinical dementia rating scale; ADSAS-Cog: Alzheimer’s Disease Assessment Scale–Cognitive Subscale; RUDAS: Rowland Universal Dementia Assessment Scale; GDS: Geriatric Depression Scale; RBANS: Repeatable Battery for the Assessment of Neuropsychological Status; ED: emergency department.

Nation	Number of Patients	Number of Televisits	Mean Disease Duration	Sex of Participants (% male)	Mean Age of Participants	Software	Healtcare Providers Involved	Addressed Issues	Feedback	Reference
**Netherlands**	151	20 per patients	2 years	29.1	57.3 (5.3)	iVitality	physician, research nurse	MMSE, 15-WVLT, TMT, Stroop-color test	smartphone-based cognitive testing seems promising for large-scale data-collection	[19]
U.S.	33 and 33 caregivers	66	2 weeks	59 (both patients and caregivers)	patients 71.6 (51–96); caregivers 65.3 (38–79)	Cisco’s Jabber TelePresence platform HIPAA compliant	research assistant, clinicians	MoCA and CDR	telemedicine is a feasible option for assessing cognitive function and caregiver coping	[14]
Italy	28	5 per patients	2 years	28.5	73.9 (7.45)	BCC950 Logitech	psychologist	MMSE, ADSAS-Cog	reliable approach to document cognitive stability or decline, and to measure treatment effects	[20]
**Australia**	90	median 6 per patients	median 42-84 weeks	33.3	79.14	Apple iPad or a Microsoft Surface Pro	clinicians, interpreter	RUDAS and GDS score	reliable as face-to-face interpreting. Cost analysis: that video-interpreting is cheaper than face-to-face interpreting	[13]
U.S.	15 (12 demented, 2 MCI and 1 HC)	15	-	100	79.1 (71–88)	Tandberg classic (older) end points and MXP (newer) end points	clinicians	neuropsychological battery	VTM is emerging as an effective way to provide consultation and care to rural residents	[21]
Australia	42 subjects (43% with cognitive deficits)	42	-	-	75 (41–95)	the units were connected via an ISDN simulator (Liberator SI/8B0P/01) at an ISDN bandwidth of 384 kbit/s	clinicians	RUDAS	RUDAS can be reliably administered via VC in post acute patients as an alternative to FTF administration	[22]
**U.S.**	17	34	-	88.2	62.8 (14.50)	Cisco TelePresence Precision HD USB Web camera utilising Cisco Jabber Video for Telepresence Software	clinicians	MoCA	MoCA is valid and reliable test also when performed using telemedicine	[23]
U.S.	18 (7 HC, 6 MCI, 5 AD)	18	-	61.1%	69.67 (58–84)	Polycom iPower 680 series videoconferencing system	clinicians	RBANS	feasibility and reliability of remote administration of the RBANS via VTC	[24]
**U.S.**	10	10	-	100%	73.9 (68–78)	TCP/IP (Transmission Control Protocol/Internet Protocol) with the h323VOP standard	licensed speech and language pathologist	Picture Description (auditory response version, Boston Diagnostic Aphasia Examination), Boston Naming Test, Token Test, Aural Comprehension of Words and Phrases, Controlled Oral Word Association Test	no significant difference	[25]
**U.S.**	22	22	-	77.3%	70.7 (9.65)	CODEC	clinicians and researcher	MMSE, Hopkins Verbal Learning Test Revised, Digit Span subtest from the Escala de Inteligencia de Wechsler para Adultos—Tercera Edicion, Letter and Category Fluency, Clock Drawing, Brief Visuospatial Memory Test and Ponton-Satz Spanish Naming Test	no significant differences between cognitive scores, depending on the testing modality	[26]
**U.S.**	197 (78 with dementia)	78	-	53.8%	72.7 (8.43 SD)	Polycom iPower 680 series videoconferencing system	clinicians	MMSE, Hopkins Verbal Learning Test-Revised, letter and category fluency, Boston Naming Test-15 item, Digit Span forward and backward, Clock Drawing Test and the Geriatric Depression Scale-15 item	online tests can distinguish between patients with and without cognitive impairment as usual face to face assessment	[12]
U.S.	35 (14 with MCI and 19 with AD)	35	1 month	72.7%	73.3 (51–84)	H.323 PC-based Videoconferencing System	clinicians	MMSE, Hopkins Verbal Learning Test–Revised, Clock Drawing Test, Digit Span, Category Fluency, letter fluency and 15-item versions of the Boston Naming Test	telecognitive assessment is a valid means to conduct neuropsychological evaluation of older adults with cognitive impairment	[27]
**U.S.**	84 (29 MCI and 55 HC)	89	-	37%	64.89 (46–88)	Polycom iPower 680 series videoconferencing system	clinicians	MMSE, Clock Drawing, Digit Span Forward and Backward, Oral Trails, Hopkins Verbal Learning Test-Revised, Letter and Category Fluency, and a short form Boston Naming Test	video teleconferencing appears a valid tool to remotely administer neuropsycological tests	[28]
Korea	427	-	5 years	28.3%	79 (60–95)	Tandberg 990	clinicians, nurses	video interviews	the treatment duration was greater than 2 years for those using the telemedicine system	[15]
**U.S.**	731	201	-	38%	86 (80–90)	videotelemedicine	clinicians	video interviews	telemedicine can decrease ED use by demented	[17]

**Table 3:** **Case series, observational studies and randomized controlled trials on telemedicine in Parkinson’s Disease.** Continuous variables are expressed as mean (SD) unless otherwise specified. § Mean (SE). Abbreviations: HBMM: home-based motor monitoring; ISIBT: in-clinic sensory integration balance training; LSVT: Lee Silverman Voice Treatment; OBM: office-based management; SRE: self-regulated exercise; TAE: telecoach-assisted exercise; TAU: treatment as usual; TC: telemedicine care; TM: telemedicine; T-CBT: telephone-based cognitive-behavioral treatment; UC: usual care; VHC: virtual house calls; VRT: virtual reality telerehabilitation. Reference period: until the end of 2019 (excluded studies involving the COVID-19 period, cited in the Section 2.2).

Nation.	Number of Patients	Study Follow-Up	Mean Disease Duration (years)	Sex of Participants (n and % male)	Mean Age of Participants (Years)	UPDRS Part III Score	Software/ Telehealth Support	Healthcare Providers Involved	Addressed Issues	Feedback	Reference
*Studies on care delivery and clinical evaluation*											
U.S.	34	3 years (100 visits)	-	-	-	-	Vcon Armada Cruiser Polycom ViewStation/Tandberg Intern MXP videoconferencing unit	movement disorder specialist, health-care provider, carer	reliability of remote UPDRS assessment, savings in travel and lodging costs, patients’ satisfaction	satisfactory motor UPDRS measurement, savings amounted to 1500 attendant travel hours, 100,000 travel kilometers, and US$37,000 in travel and lodging costs, satisfaction of patients and providers	[33]
U.S.	10 -TC: *n* = 6 -UC: *n* = 4	6 months (3 visits)	-	-TC: 4 (66.7%) -UC (0)	-TC: 71.7 (7.9) -UC: 69.8 (7.0)	-TC: 29.8 (8.6) -UC: 34.0 (21.4)	VSee Videoconferencing and Polycom software	movement disorder specialist	feasibility, changes in quality of life, satisfaction, motor performance, mood, cognition	significant improvements in quality of life and motor performance	[40]
Germany	78	30 days	9.7 (0.6)	44 (56.4%)	67 (8)	31.2 (8.9)	MVB—Medizinische Videobeobachtung GmbH	movement disorder specialist	patients sent video recordings made in the home to the treating team via the Internet	patients and a blind rater rated their PD as improved from baseline	[41]
U.S.	20 -TC: 9 -UC: 11	7 months (27 visits)	-	-TC: 77.8% -UC: 72.7%	-TC: 66.6 (4.0) § -UC: 64.5 (3.4) §	-TC: 27.1 (3.0) § -UC: 27.7 (2.5) §	Vidyo videoconferencing software	movement disorder specialist	feasibility, clinical benefit, economic value	change in quality of life not different in TC group; TM visits saved participant an average of 100 miles of travel and 3 h of time	[36]
Canada	137 -34 TM users -103 TM non-users	TM users since 2009; non-users seen during October and November 2013	users: 14.5 (6.7) Non-users: 10.7 (5.9)	users: 27 (79%) Non-users: 66 (64%)	users: 65.8 (11.5) Non-users: 67.6 (9.1)	users: 24.2 Non-users: 20.7	Ontario Telemedicine Network	telehealth center (physicians and nurses)	satisfaction with telehealth, rates of patient retention, reasons for discontinuing TM, proportion of patients interested in TM	experience of support staff is an important source of dissatisfaction; most users continued with TM	[42]
U.S.	40 -20 HBMM -20 OBM	1 year	-	-HBMM: 10 (50) -OBM: 8 (40)	-HBMM: 66.44 (7.09) – OBM: 66.05 (9.76)	-HBMM: 28.93 (12.81) -OBM: 33.75 (10.79)	Kinesia^TM^ (tablet software app, wireless finger-worn motion sensor unit, automated web-based symptom reporting)	-	motor and non-motor symptom severities, quality of life, neuropsychiatric symptoms, comorbidities; Cost-effectiveness	UPDRS parts I, III, IV and quality-adjusted life-years scores similar between both groups. HBMM was cost-effective in terms of improvement of functional status, motor severity, and motor complications	[35]
U.S.	195 -VHC: 97 -UC: 98	1 year (4 visits)	-VHC: 8.3 (6.15) -UC: 7.6 (4.9)	-VHC: 49 (50.5%) -UC: 42 (42.8%)	-VHC: 65.9 (7.8) -UC: 66.9 (8.5)	-VHC: 29.5 (10.2) -UC: 28.3 (9.9)	Vidyo videoconferencing software	neurologist	feasibility, efficacy, quality of care, caregiver burden, time travel savings	VHC was neither more nor less efficacious than usual in-person care. VHC generated great interest and provided substantial convenience	[37]
Italy	398	March 12th - May 14th, 2020 (194 video-consultations)	8.6 (6.3)	59.1%	73.7 (9.7)	-	Zoom^®^ and Microsoft Teams^®^ platforms	PD nurse specialists, neurologistis, multidisciplinary team	Evaluation of a two-step telehealth model based on a telenursing triage followed by video-consultations by experienced neurologists	Feasibility of the two-step approach; reimbursement by the Lombardy Regional health system	[57]
*Studies on speech therapy*											
U.K.	3	16 h/four times per week for four weeks	3-6	3 (100%)	63–72	-	Skype^TM^	speech therapist	online speech therapy with the LSVT	similar treatment gains between subjects treated over the Internet and those treated face to face	[45]
Australia	61	31 assessments face-to-face, 30 led online	6.52 (6.53)	42 (68.8%)	69.23 (8.60)	-	videoconferencing link via the Internet	two speech–language pathologists	online speech therapy with the LSVT	online assessment appears to be valid and reliable	[48]
U.K.	29 -8 iPad LSVT -21 in-person	18 sessions	-	-	-iPad LSVT: 67 (6.05) -in-person treatment: 69 years (7.98)	-	iPad-based ‘Facetime’	speech therapist	comparison between treatment with conventional ‘in person’ LSVT and those treated remotely	iPad LSVT is non-inferior compared to conventional treatment	[46]
Australia	8	two 90-min sessions per week for 4 weeks	4.50 (1.51)	6 (75%)	68.50 (8.28)	-	Adobe connect	speech therapist	feasibility of delivering a group speech maintenance programme	significant improvements for all sound pressure level measures	[47]
*Studies on physical therapy*											
Italy	76 -VRT: 38 -ISIBT: 38	21 sessions 50 min each for 7 consecutive weeks	-VRT: 6.16 (3.81) -ISIBT: 7.47 (3.90)	-VRT: 23 (60.5%) -ISIBT: 28 (73.6%)	-VRT: 67.45 (7.18) -ISIBT: 69.84 (9.41)	-VRT: 44.13 (24.05) -ISIBT: 50.76 (24.12)	Skype^TM^	two physiotherapists	improvements in postural stability after remotely supervised in-home VRT and ISIBT	VR is a feasible alternative to in-clinic SIBT	[49]
U.S.	20 -10 TAE -10 SRE	8 weeks of exercise (3 sessions per week: 24 total sessions)	-TAE: 6.55 (4.52) -SRE: 7.55 (4.78)	-TAE: 7 (70%) -SRE: 7 (70%)	-TAE: 63.4 (10.4) -SRE: 70.8 (7.1)	-	Android computer tablet with Bluetooth and wireless Internet capability	physical therapist	uptake and implementation of two common methods of Internet exercise training	TAE participants achieved stronger attendance compared to SRE participants	[50]
*Studies on cognitive behavioral therapy*											
U.S.	20	10-weeks + 14 weeks follow-up	7.45 (5.17)	8 (38.10%)	65.86 (9.32)	-	telephone			phone-based therapy was a feasible and helpful approach	[65]
U.S.	72	10-session T-CBT (6 months)	-T-CBT: 6.95 (7.82) -TAU: 5.65 (4.20)	-T-CBT: 17 (23.61) -TAU: 18 (25.00)	-T-CBT: 65.62 (9.76) -TAU: 65.62 (9.76)	-	telephone	neurologistspsychiatriststherapists	efficacy of T-CBT intervention for depression compared to TAU	Hamilton Depression Rating Scale score improved significantly in T-CBT compared to TAU	[52]

**Table 4:** **Telemedicine studies in Amyotrophic Lateral Sclerosis.** Continuous variables are expressed as mean (SD or confidence interval), unless otherwise specified. RRD: Roessingh Research and Development; VPN: virtual private network; ER: emergency room; NIV: non-invasive ventilation

Nation	Number of Patients	Number of Televisits	Mean Disease Duration	Sex of Participants (% male)	Mean Age of Participants	Software	Healtcare Providers Involved	Addressed Issues	Feedback	Reference
Netherlands	4	-	-	75%	42 (35–57)	RRD and VPN	physician	symptoms, treatment options, progress, palliative	feasible for discussing pratical issues, not for psychological and emotional issues	[68]
U.S.	32	-	32 months	100%	63.03 (+/− 15.26)	clinical video telehealth; Video-to-home	neurologist + nurse (+ ohers based on patients needs)	care management	patients managed by telemedicine received the same quality of care and had similar outcomes to those patients seen via face-to-face encounters	[71]
U.S.	97	136	30 months	63%	58 (29–89)	HIPAA-compliant	4 physicians; 1 nurse	medication management, discussion of goals of care, research, equipment uses	feasible for supplement of traditional multidisciplinary ALS care	[69]
U.S.	35 → 18	27	52 months	66%	64 (39–79)	iPad	multidisciplinary team	store and forward method for telemedicine (patients assessed by a single trained individual in their home)	excellent satisfaction	[88]
*Studies on ventilatory functions*										
Italy	40	5/month for 8.6 months	-	60%	63 (+/− 11)	telephone	pneumologist, neurologist, nurse, psychologist	respiratory status monitoring	extremely satisfied	[76] (expanded in [77])
Portugal	40	-	362 days	60%	62 (12.90)	modem device	-	adaption of and testing a modem NIV communication devices	the number of ER visits and in-hospital admissions was significantly lower in group monitoring with modem	[78]
UK	13	136 → 61 intervention	14	61.5%	66	Careportal	physician	development of self-reported questions for telemonitoring in patients with NIV	maintenance of ventilation and SpO2 levels despite illness progression	[80]
*Studies during the COVID-19 pandemic*										
Italy	19	310	10 months	37%	51 (+/− 12)	TiCuro platfrom	multidisciplinary team	to reproduce the multidisciplinary approach by telemedicine	stabilization of the functional and metabolic status and improvement of the psychological one	[85]
Italy	32	30	7.4 months	-	65 (+/− 9.5)	phone	physician	monitoring during the pandemic	valid tool to triage patients	[83]
Italy	139	139	21 months	51%	67 (59–74)	mostly phone	Neurologist + psychologist	monitoring during the pandemic	satisfied but the majority prefer a face-to-face visit	[84]

## Data Availability

No new data were created or analyzed in this study. Data sharing is not applicable to this article.

## References

[1] Erkkinen M.G., Kim M.-O., Geschwind M.D. (2018). Clinical Neurology and Epidemiology of the Major Neurodegenerative Diseases. Cold Spring Harb. Perspect. Biol..

[2] Wyss-Coray T. (2016). Ageing, neurodegeneration and brain rejuvenation. Nat. Cell Biol..

[3] Hou Y., Dan X., Babbar M., Wei Y., Hasselbalch S.G., Croteau D.L., Bohr V.A. (2019). Ageing as a risk factor for neurodegenerative disease. Nat. Rev. Neurol..

[4] Jia J., Wei C., Chen S., Li F., Tang Y., Qin W., Zhao L., Jin H., Xu H., Wang F. (2018). The cost of Alzheimer’s disease in China and re-estimation of costs worldwide. Alzheimer Dement..

[5] Rodriguez-Blazquez C., Forjaz M.J., Tudela L.L., Paz S., Martínez-Martín P. (2015). Estimating the direct and indirect costs associated with Parkinson’s disease. Expert Rev. Pharm. Outcomes Res..

[6] Oh J., An J.W., Oh S.-I., Oh K.W., A Kim J., Lee J.S., Kim S.H. (2015). Socioeconomic costs of amyotrophic lateral sclerosis according to staging system. Amyotroph. Lateral Scler. Front. Degener..

[7] Wake E., Atkins H., Willock A., Hawkes A., Dawber J., A Weir K. (2020). Telehealth in trauma: A scoping review. J. Telemed. Telecare.

[8] Bashshur R.L. (1995). On the Definition and Evaluation of Telemedicine. Telemed. J..

[9] Patterson C. (2018). World Alzheimer Report 2018—The State of the Art of Dementia Research: New Frontiers.

[10] Geddes M.R., O’Connell M.E., Fisk J.D., Gauthier S., Camicioli R., Ismail Z. (2020). Remote cognitive and behavioral assessment: Report of the Alzheimer Society of Canada Task Force on dementia care best practices for COVID-19. Alzheimer’s Dementia: Diagn. Assess. Dis. Monit..

[11] Loh P.-K., Ramesh P., Maher S., Saligari J., Flicker L., Goldswain P. (2004). Can patients with dementia be assessed at a distance? The use of Telehealth and standardised assessments. Intern. Med. J..

[12] Wadsworth H.E., Dhima K., Womack K.B., Hart J., Weiner M.F., Hynan L.S., Cullum C.M. (2018). Validity of Teleneuropsychological Assessment in Older Patients with Cognitive Disorders. Arch. Clin. Neuropsychol..

[13] Hwang K., De Silva A., A Simpson J., Logiudice D., Engel L., Gilbert A.S., Croy S., Haralambous B. (2020). Video-interpreting for cognitive assessments: An intervention study and micro-costing analysis. J. Telemed. Telecare.

[14] Lindauer A., Seelye A., Lyons B., Dodge H.H., Mattek N., Mincks K., Kaye J., Erten-Lyons D. (2017). Dementia Care Comes Home: Patient and Caregiver Assessment via Telemedicine. Gerontology.

[15] Cheong C.-K., Lim K.-H., Jang J.-W., Jhoo J.H. (2015). The effect of telemedicine on the duration of treatment in dementia patients. J. Telemed. Telecare.

[16] LaMantia M.A., Stump T.E., Messina F.C., Miller D.K., Callahan C.M. (2016). Emergency Department Use Among Older Adults With Dementia. Alzheimer Dis. Assoc. Disord..

[17] Gillespie S.M., Wasserman E.B., Wood N.E., Wang H., Dozier A., Nelson D., McConnochie K.M., Shah M.N. (2019). High-Intensity Telemedicine Reduces Emergency Department Use by Older Adults With Dementia in Senior Living Communities. J. Am. Med. Dir. Assoc..

[18] Williams K.N., Shaw C.A., Perkhounkova Y., Hein M., Coleman C.K. (2020). Satisfaction, utilization, and feasibility of a telehealth intervention for in-home dementia care support: A mixed methods study. Dementia.

[19] Jongstra S., Wijsman L.W., Cachucho R., Hoevenaar-Blom M.P., Mooijaart S., Richard E. (2017). Cognitive Testing in People at Increased Risk of Dementia Using a Smartphone App: The iVitality Proof-of-Principle Study. JMIR mHealth uHealth.

[20] Carotenuto A., Rea R., Traini E., Ricci G., Fasanaro A.M., Amenta F. (2018). Cognitive Assessment of Patients With Alzheimer’s Disease by Telemedicine: Pilot Study. JMIR Ment. Health.

[21] Barton C., Morris R., Rothlind J., Yaffe K. (2011). Video-Telemedicine in a Memory Disorders Clinic: Evaluation and Management of Rural Elders with Cognitive Impairment. Telemed. E Health.

[22] Wong L., Martin-Khan M., Rowland J., Varghese P., Gray L.C. (2012). The Rowland Universal Dementia Assessment Scale (RUDAS) as a reliable screening tool for dementia when administered via videoconferencing in elderly post-acute hospital patients. J. Telemed. Telecare.

[23] DeYoung N., Shenal B.V. (2019). The reliability of the Montreal Cognitive Assessment using telehealth in a rural setting with veterans. J. Telemed. Telecare.

[24] Galusha-Glasscock J.M., Horton D.K., Weiner M.F., Cullum C.M. (2016). Video Teleconference Administration of the Repeatable Battery for the Assessment of Neuropsychological Status. Arch. Clin. Neuropsychol..

[25] Vestal L., Smith-Olinde L., Hicks G., Hutton T., Hart J. (2006). Efficacy of language assessment in Alzheimer’s disease: Comparing in-person examination and telemedicine. Clin. Interv. Aging.

[26] Vahia I.V., Ng B., Camacho A., Cardenas V., Cherner M., Depp C.A., Palmer B.W., Jeste D.V., Agha Z. (2015). Telepsychiatry for Neurocognitive Testing in Older Rural Latino Adults. Am. J. Geriatr. Psychiatry Off. J. Am. Assoc. Geriatr. Psychiatry.

[27] Cullum C.M., Weiner M.F., Gehrmann H.R., Hynan L.S. (2006). Feasibility of Telecognitive Assessment in Dementia. Assessment.

[28] Wadsworth H.E., Galusha-Glasscock J.M., Womack K.B., Quiceno M., Weiner M.F., Hynan L.S., Shore J., Cullum C.M. (2016). Remote Neuropsychological Assessment in Rural American Indians with and without Cognitive Impairment. Arch. Clin. Neuropsychol..

[29] Sethi K.D. (2002). Clinical aspects of Parkinson disease. Curr. Opin. Neurol..

[30] Ferreira J.J., Godinho C., Santos A.T., Domingos J.M.M., Abreu D., Lobo R., Gonçalves N., Barra M., Larsen F., Fagerbakke Ø. (2015). Quantitative home-based assessment of Parkinson’s symptoms: The SENSE-PARK feasibility and usability study. BMC Neurol..

[31] Ramsperger R., Meckler S., Heger T., Van Uem J., Hucker S., Braatz U., Graessner H., Berg D., Manoli Y., Serrano J.A. (2016). Continuous leg dyskinesia assessment in Parkinson’s disease –clinical validity and ecological effect. Park. Relat. Disord..

[32] Heldman D.A., Giuffrida J.P., Cubo E. (2016). Wearable Sensors for Advanced Therapy Referral in Parkinson’s Disease. J. Park. Dis..

[33] Samii A., Ryan-Dykes P., Tsukuda R.A., Zink C., Franks R., Nichol W.P. (2006). Telemedicine for delivery of health care in Parkinson’s disease. J. Telemed. Telecare.

[34] Abdolahi A., Scoglio N., Killoran A., Dorsey E.R., Biglan K.M. (2013). Potential reliability and validity of a modified version of the Unified Parkinson’s Disease Rating Scale that could be administered remotely. Park. Relat. Disord..

[35] Cubo E., Mariscal N., Solano B., Becerra V., Armesto D., Calvo S., Arribas J., Seco J., Martinez A., Zorrilla L. (2016). Prospective study on cost-effectiveness of home-based motor assessment in Parkinson’s disease. J. Telemed. Telecare.

[36] Dorsey E.R., Venkataraman V., Grana M.J., Bull M.T., George B.P., Boyd C.M., Beck C.A., Rajan B., Seidmann A., Biglan K.M. (2013). Randomized controlled clinical trial of “virtual house calls” for Parkinson disease. JAMA Neurol..

[37] Beck C.A., Beran D.B., Biglan K.M., Boyd C.M., Dorsey E.R., Schmidt P.N., Simone R., Willis A.W., Galifianakis N.B., Katz M. (2017). National randomized controlled trial of virtual house calls for Parkinson disease. Neurology.

[38] Durner G., Durner J., Dunsche H., Walle E., Kurzreuther R., Handschu R. (2016). 24/7 Live Stream Telemedicine Home Treatment Service for Parkinson’s Disease Patients. Mov. Disord. Clin. Pract..

[39] Biglan K.M., Voss T.S., Deuel L.M., Miller D., Eason S., Fagnano M., George B.P., Appler A., Polanowicz J., Viti L. (2009). Telemedicine for the care of nursing home residents with Parkinson’s disease. Mov. Disord..

[40] Dorsey E.R., Deuel L.M., Voss T.S., Finnigan K., George B.P., Eason S., Miller D., Reminick J.I., Appler A., Polanowicz J. (2010). Increasing access to specialty care: A pilot, randomized controlled trial of telemedicine for Parkinson’s disease. Mov. Disord..

[41] Marzinzik F., Wahl M., Doletschek C.M., Jugel C., Rewitzer C., Klostermann F. (2012). Evaluation of a telemedical care programme for patients with Parkinson’s disease. J. Telemed. Telecare.

[42] Qiang J., Marras C. (2015). Telemedicine in Parkinson’s disease: A patient perspective at a tertiary care centre. Park. Relat. Disord..

[43] Dorsey E.R., Achey M.A., Beck C.A., Beran D.B., Biglan K.M., Boyd C.M., Schmidt P.N., Simone R., Willis A.W., Galifianakis N.B. (2016). National Randomized Controlled Trial of Virtual House Calls for People with Parkinson’s Disease: Interest and Barriers. Telemed. E Health.

[44] Spear K.L., Auinger P., Simone R., Dorsey E.R., Francis J. (2019). Patient Views on Telemedicine for Parkinson Disease. J. Park. Dis..

[45] Howell S., Tripoliti E., Pring T. (2009). Delivering the Lee Silverman Voice Treatment (LSVT) by web camera: A feasibility study. Int. J. Lang. Commun. Disord..

[46] Griffin M., Bentley J., Shanks J., Wood C. (2018). The effectiveness of Lee Silverman Voice Treatment therapy issued interactively through an iPad device: A non-inferiority study. J. Telemed. Telecare.

[47] Quinn R., Park S., Theodoros D., Hill A.J. (2018). Delivering group speech maintenance therapy via telerehabilitation to people with Parkinson’s disease: A pilot study. Int. J. Speech Lang. Pathol..

[48] A Constantinescu G., Theodoros D., Russell T., Ward E., Wilson S., Wootton R. (2010). Assessing disordered speech and voice in Parkinson’s disease: A telerehabilitation application. Int. J. Lang. Commun. Disord..

[49] Gandolfi M., Geroin C., Dimitrova E., Boldrini P., Waldner A., Bonadiman S., Picelli A., Regazzo S., Stirbu E., Primon D. (2017). Virtual Reality Telerehabilitation for Postural Instability in Parkinson’s Disease: A Multicenter, Single-Blind, Randomized, Controlled Trial. BioMed Res. Int..

[50] Lai B., Bond K., Kim Y., Barstow B., Jovanov E., Bickel C.S. (2018). Exploring the uptake and implementation of tele-monitored home-exercise programmes in adults with Parkinson’s disease: A mixed-methods pilot study. J. Telemed. Telecare.

[51] Veazey C., Cook K.F., Stanley M., Lai E.C., Kunik M.E. (2009). Telephone-Administered Cognitive Behavioral Therapy: A Case Study of Anxiety and Depression in Parkinson’s Disease. J. Clin. Psychol. Med. Settings.

[52] Dobkin R.D., Mann S.L., Gara M.A., Interian A., Rodriguez K.M., Menza M. (2020). Telephone-based cognitive behavioral therapy for depression in Parkinson disease: A randomized controlled trial. Neurology.

[53] Seritan A.L., Heiry M., Iosif A.-M., Dodge M., Ostrem J.L. (2019). Telepsychiatry for patients with movement disorders: A feasibility and patient satisfaction study. J. Clin. Mov. Disord..

[54] Willows T., Dizdar N., Nyholm D., Widner H., Grenholm P., Schmiauke U., Urbom A., Groth K., Larsson J., Permert J. (2017). Initiation of Levodopa-Carbidopa Intestinal Gel Infusion Using Telemedicine (Video Communication System) Facilitates Efficient and Well-Accepted Home Titration in Patients with Advanced Parkinson’s Disease. J. Park. Dis..

[55] Jitkritsadakul O., Rajalingam R., Toenjes C., Munhoz R.P., Fasano A. (2018). Tele-health for patients with deep brain stimulation: The experience of the Ontario Telemedicine Network. Mov. Disord..

[56] Pérez-López C., Samà A., Rodríguez-Martín D., Català A., Cabestany J., Moreno-Arostegui J.M., De Mingo E., Rodríguez-Molinero A. (2015). Remote control of apomorphine infusion rate in Parkinson’s disease: Real-time dose variations according to the patients’ motor state. A proof of concept. Park Relat. Disord..

[57] Papa S.M., Brundin P., Fung V.S.C., Kang U.J., Burn D.J., Colosimo C., Chiang H.-L., Alcalay R.N., Trenkwalder C., MDS-Scientific Issues Committee (2020). Impact of the COVID-19 pandemic on Parkinson’s disease and movement disorders. Mov. Disord..

[58] Cilia R., Mancini F., Bloem B.R., Eleopra R. (2020). Telemedicine for parkinsonism: A two-step model based on the COVID-19 experience in Milan, Italy. Park. Relat. Disord..

[59] Fasano A., Antonini A., Katzenschlager R., Krack P., Odin P., Evans A.H., Foltynie T., Volkmann J., Merello M. (2020). Management of Advanced Therapies in Parkinson’s Disease Patients in Times of Humanitarian Crisis: The COVID -19 Experience. Mov. Disord. Clin. Pract..

[60] Miocinovic S., Ostrem J.L., Okun M.S., Bullinger K.L., Riva-Posse P., Gross R.E., Buetefisch C.M. (2020). Recommendations for Deep Brain Stimulation Device Management During a Pandemic. J. Park. Dis..

[61] Zhang C., Zhu K., Lin Z., Huang P., Pan Y., Sun B., Li D. (2020). Utility of Deep Brain Stimulation Telemedicine for Patients With Movement Disorders During the COVID -19 Outbreak in China. Neuromodulation Technol. Neural Interface.

[62] Quinn L., MacPherson C., Long K., Shah H. (2020). Promoting Physical Activity via Telehealth in People With Parkinson Disease: The Path Forward After the COVID-19 Pandemic?. Phys. Ther..

[63] Srivastav A.K., Samuel A.J. (2020). E-Rehabilitation: One solution for patients with Parkinson’s disease in COVID-19 era. Park. Relat. Disord..

[64] Mulroy E., Menozzi E., Lees A.J., Lynch T., Lang A.E., Bhatia K.P. (2020). Telemedicine in Movement Disorders: Leçons du COVID-19. Mov. Disord..

[65] Dobkin R.D., Menza M., Allen L.A., Tiu J., Friedman J., Bienfait K.L., Gara M.A., Mark M.H. (2011). Telephone-Based Cognitive–Behavioral Therapy for Depression in Parkinson Disease. J. Geriatr. Psychiatry Neurol..

[66] Hobson E.V., Baird W.O., Cooper C.L., Mawson S., Shaw P.J., Mcdermott C.J. (2016). Using technology to improve access to specialist care in amyotrophic lateral sclerosis: A systematic review. Amyotroph. Lateral Scler. Front. Degener..

[67] Paganoni S., Nicholson K., Leigh F., Swoboda K.J., Chad D., Drake K., Haley K., Cudkowicz M., Berry J.D. (2017). Developing multidisciplinary clinics for neuromuscular care and research. Muscle Nerve.

[68] Nijeweme-d’Hollosy W.O., Janssen E.P.F., Huis in ’t Veld R.M.H.A., Spoelstra J., Vollenbroek-Hutten M.M.R., Hermens H.J. (2006). Tele-treatment of patients with amyotrophic lateral sclerosis (ALS). J. Telemed. Telecare.

[69] Van De Rijn M., Paganoni S., Levine-Weinberg M., Campbell K., Swartz Ellrodt A., Estrada J., Cohen A.B., Schwamm L.H., Berry J.D. (2018). Experience with telemedicine in a multi-disciplinary ALS clinic. Amyotroph. Lateral Scler. Front. Degener..

[70] Paganoni S., Van De Rijn M., Drake K., Burke K., Doyle M., Dpt A.S.E., Nicholson K., Atassi N., De Marchi F., Babu S. (2019). Adjusted cost analysis of video televisits for the care of people with amyotrophic lateral sclerosis. Muscle Nerve.

[71] Selkirk S.M., Washington M.O., McClellan F., Flynn B., Seton J.M., Strozewski R. (2017). Delivering tertiary centre specialty care to ALS patients via telemedicine: A retrospective cohort analysis. Amyotroph. Lateral Scler. Front. Degener..

[72] Amato S., Poliandri G., Sgroi D., Clarici L., De Salazar V. (2020). [Telemedicine and improvement in care quality. The experience of Rome 3 Healthcare Local Autority in home care of patients with amyotrophic lateral sclerosis (ALS)]. Ig. Sanita Pubbl..

[73] Hobson E.V., Baird W.O., Partridge R., Cooper C.L., Mawson S., Quinn A., Shaw P.J., Walsh T., Wolstenholme D., Mcdermott C.J. (2018). The TiM system: Developing a novel telehealth service to improve access to specialist care in motor neurone disease using user-centered design. Amyotroph. Lateral Scler. Front. Degener..

[74] Helleman J., Van Eenennaam R., Kruitwagen E.T., Kruithof W.J., Slappendel M.J., Berg L.H.V.D., Visser-Meily J.M., Beelen A. (2020). Telehealth as part of specialized ALS care: Feasibility and user experiences with “ALS home-monitoring and coaching”. Amyotroph. Lateral Scler. Front. Degener..

[75] Wills A.M., Garry J., Hubbard J., Mezoian T., Breen C.T., Ortiz-Miller C., Nalipinski P., Sullivan S., Berry J.D., Cudkowicz M.E. (2019). Nutritional counseling with or without mobile health technology: A randomized open-label standard-of-care-controlled trial in ALS. BMC Neurol..

[76] Vitacca M., Comini L., Tentorio M., Assoni G., Trainini D., Fiorenza D., Morini R., Bruletti G., Scalvini S. (2010). A pilot trial of telemedicine-assisted, integrated care for patients with advanced amyotrophic lateral sclerosis and their caregivers. J. Telemed. Telecare.

[77] Vitacca M., Comini L., Assoni G., Fiorenza D., Gilè S., Bernocchi P., Scalvini S. (2012). Tele-assistance in patients with amyotrophic lateral sclerosis: Long term activity and costs. Disabil. Rehabilitation: Assist. Technol..

[78] Pinto A.C., Almeida J.P., Pinto S.C., Pereira J., Oliveira A.G., De Carvalho M. (2010). Home telemonitoring of non-invasive ventilation decreases healthcare utilisation in a prospective controlled trial of patients with amyotrophic lateral sclerosis. J. Neurol. Neurosurg. Psychiatry.

[79] de Almeida J.P.L., Pinto A.C., Pereira J., Pinto S., de Carvalho M. (2010). Implementation of a wireless device for real-time telemedical assistance of home-ventilated amyotrophic lateral sclerosis patients: A feasibility study. Telemed E Health.

[80] Cousins R., Ashcroft-Kelso H., Halhead R., Young C., Chakrabarti B., Levene P., Cousins R., Angus R.M. (2019). Incorporating self-reported questions for telemonitoring to optimize care of patients with MND on noninvasive ventilation (MND OptNIVent). Amyotroph. Lateral Scler. Front. Degener..

[81] Bombaci A., Abbadessa G., Trojsi F., Leocani L., Bonavita S., Lavorgna L. (2021). Telemedicine for management of patients with amyotrophic lateral sclerosis through COVID-19 tail. Neurol. Sci..

[82] Andrews J.A., Berry J.D., Baloh R.H., Carberry N., Cudkowicz M.E., Dedi B., Glass J., Maragakis N.J., Miller T.M., Paganoni S. (2020). Amyotrophic Lateral Sclerosis Care and Research in the USA During the COVID-19 Pandemic: Challenges and Opportunities. Muscle Nerve.

[83] Capozzo R., Zoccolella S., Musio M., Barone R., Accogli M., Logroscino G. (2020). Telemedicine is a useful tool to deliver care to patients with Amyotrophic Lateral Sclerosis during COVID-19 pandemic: Results from Southern Italy. Amyotroph. Lateral Scler. Front. Degener..

[84] Vasta R., Moglia C., D’Ovidio F., Di Pede F., De Mattei F., Cabras S., Peotta L., Iazzolino B., Giusiano S., Manera U. (2020). Telemedicine for patients with amyotrophic lateral sclerosis during COVID-19 pandemic: An Italian ALS referral center experience. Amyotroph. Lateral Scler. Front. Degener..

[85] De Marchi F., Sarnelli M.F., Serioli M., De Marchi I., Zani E., Bottone N., Ambrosini S., Garone R., Cantello R., Mazzini L. (2020). Telehealth approach for amyotrophic lateral sclerosis patients: The experience during COVID-19 pandemic. Acta Neurol. Scand..

[86] De Marchi F., Cantello R., Ambrosini S., Mazzini L., Group C.S. (2020). Telemedicine and technological devices for amyotrophic lateral sclerosis in the era of COVID-19. Neurol. Sci..

[87] Manera U., Cabras S., Daviddi M., Vasta R., Torrieri M.C., Palumbo F., Bombaci A., Grassano M., Solero L., Peotta L. (2020). Validation of the Italian version of self-administered ALSFRS-R scale. Amyotroph. Lateral Scler. Front. Degener..

[88] Pulley M.T., Brittain R., Hodges W., Frazier C., Miller L., Matyjasik-Liggett M., Maurer S., Peters M., Solomon K., Berger A.R. (2019). Multidisciplinary amyotrophic lateral sclerosis telemedicine care: The store and forward method. Muscle Nerve.

[89] Bassan S. (2020). Data privacy considerations for telehealth consumers amid COVID-19. J. Law Biosci..

[90] Raposo V.L. (2016). Telemedicine: The legal framework (or the lack of it) in Europe. GMS Health Technol Assess.

[91] Grossman S.N., Han S.C., Balcer L.J., Kurzweil A., Weinberg H., Galetta S.L., Busis N.A. (2020). Rapid implementation of virtual neurology in response to the COVID-19 pandemic. Neurology.

[92] Govindarajan R., Berry J.D., Paganoni S., Pulley M.T., Simmons Z. (2020). Optimizing telemedicine to facilitate amyotrophic lateral sclerosis clinical trials. Muscle Nerve.

